# Wrist ballistocardiography and invasively recorded blood pressure in healthy volunteers during reclining bike exercise

**DOI:** 10.3389/fphys.2023.1189732

**Published:** 2023-05-12

**Authors:** Torjus L. Steffensen, Filip E. Schjerven, Hans M. Flade, Idar Kirkeby-Garstad, Emma Ingeström, Fredrik S. Solberg, Martin Steinert

**Affiliations:** ^1^ Department of Circulation and Medical Imaging, Norwegian University of Science and Technology, Trondheim, Norway; ^2^ Department of Computer Science, Norwegian University of Science and Technology, Trondheim, Norway; ^3^ St. Olav’s University Hospital, Trondheim, Norway; ^4^ Department of Mechanical Engineering, Stanford University, Palo Alto, CA, United States; ^5^ Department of Mechanical Engineering, Norwegian University of Science and Technology, Trondheim, Norway

**Keywords:** cardiovascular monitoring, exercise physiology, cardiovascular physiology, ballistocardiography, postural effects, wearable sensors, blood pressure, noninvasive monitoring

## Abstract

**Objective:** Ballistocardiogram (BCG) features are of interest in wearable cardiovascular monitoring of cardiac performance. We assess feasibility of wrist acceleration BCG during exercise for estimating pulse transit time (PTT), enabling broader cardiovascular response studies during acute exercise and improved monitoring in individuals at risk for cardiovascular disease (CVD). We also examine the relationship between PTT, blood pressure (BP), and stroke volume (SV) during exercise and posture interventions.

**Methods:** 25 participants underwent a bike exercise protocol with four incremental workloads (0 W, 50 W, 100 W, and 150 W) in supine and semirecumbent postures. BCG, invasive radial artery BP, tonometry, photoplethysmography (PPG) and echocardiography were recorded. Ensemble averages of BCG signals determined aortic valve opening (AVO) timings, combined with peripheral pulse wave arrival times to calculate PTT. We tested for significance using Wilcoxon signed-rank test.

**Results:** BCG was successfully recorded at the wrist during exercise. PTT exhibited a moderate negative correlation with systolic BP (ρ_Sup_ = −0.65, ρ_SR_ = −0.57, ρ_All_ = −0.54). PTT differences between supine and semirecumbent conditions were significant at 0 W and 50 W (*p* < 0.001), less at 100 W (*p* = 0.0135) and 150 W (*p* = 0.031). SBP and DBP were lower in semirecumbent posture (*p* < 0.01), while HR was slightly higher. Echocardiography confirmed association of BCG features with AVO and indicated a positive relationship between BCG amplitude and SV (ρ = 0.74).

**Significance:** Wrist BCG may allow convenient PTT and possibly SV tracking during exercise, enabling studies of cardiovascular response to acute exercise and convenient monitoring of cardiovascular performance.

## 1 Introduction

Cardiovascular responses to physical exercise, particularly acute blood pressure (BP) response, are risk markers for development of hypertension and overall cardiovascular events (Miyai et al., 2002; Thanassoulis et al., 2012). Changes in pulse transit time (PTT), a correlate of arterial stiffness, between exercise and recovery have been linked to cardiovascular disease (CVD) risk factors (Liu et al., 2014), suggesting a convenient tracking exercise PTT trends could facilitate early detection or out-of-clinic monitoring of CVD patients. PTT has also garnered research interest for its potential application in cuffless BP trend tracking (Geddes et al., 1981; Kim et al., 2015; Finnegan et al., 2021). Conventional PTT recording compares the delay between a central cardiac event and the arrival of the associated waveform at a distal site; a robust method of measuring PTT by distal measurements alone would enable highly convenient monitoring of arterial stiffness.

Wearable ballistocardiography is one approach to achieve this. The ballistocardiogram (BCG) results from the mechanical force transmitted through the body with the movement of blood through the heart and major arteries (Gordon, 1877; Starr et al., 1939). The primary component of the BCG, a complex of three distinctive, energetic deflections denoted I, J, and K, is associated with systole (Rappaport, 1956; Inan et al., 2015; Tavakolian et al., 2022). Recent BCG measurement developments aim for ubiquitous longitudinal cardiovascular monitoring, for example, heart rate (HR) monitoring (Jung et al., 2021; Czapanskiy et al., 2022). Amplitude and peak width of the IJK components have been associated with stroke volume (SV) and ejection duration (Starr, 1955; Inan et al., 2015; 2009b; Ashouri et al., 2016; Quesada et al., 2021). BCG morphological variation between individuals has limited its use, but mechanistic modeling of the waveform’s complex origins is ongoing (Marazzi et al., 2022; Zaid et al., 2022).

Distally recorded BCG can be used to obtain a reasonable marker of aortic valve opening (AVO) by detecting the initial deflection of the I-wave, which occurs soon after the onset of rapid ejection. When combined with a distal marker of pulse wave arrival, such as photoplethysmography (PPG) or tonometry, this allows PTT calculation at a single distal location, enabling convenient recording with wearable sensors on distal limbs (Campo et al., 2017; Shin et al., 2022). Although a few applications of instrumented shoes have attempted to record the full-body force BCG (Liu et al., 2017; Gjerde et al., 2022), wearable ballistocardiography typically relies on accelerometry. Wrist-based accelerometers have shown the capability of detecting AVO at rest (Wiens et al., 2017), but the underlying physical mechanisms behind BCG morphology in the wrist are poorly understood (Kim et al., 2016; Yousefian et al., 2019; Shin et al., 2022).

There is limited research on wearable ballistocardiography during dynamic exercise. Morphology changes were noted in chest vibrations recorded during mild physical activity (Shandhi et al., 2021), and several studies have looked at BCG immediately post exercise (Inan et al., 2009a; Ashouri et al., 2016). Obtaining usable signals amidst severe motion artifacts presents a major challenge. However, as intensity increases, SV rises alongside HR, boosting cardiac output before plateauing with further increases driven by HR (Laughlin, 1999). We hypothesize that the systolic complex during physical activity may become more pronounced and distinguishable from motion artifacts associated with wrist BCG, given a reasonably stationary arm posture.

To enable new modalities of convenient wearable cardiovascular monitoring, our study aims to demonstrate the feasibility of wrist BCG measurements at comparatively high exercise intensity levels and to derive physiologically meaningful measures from them. We hypothesize that wrist BCG, segmented by tonometry, can be used as an AVO marker during exercise. We also investigate the relationship between BCG-PPG PTT and BP, as well as wrist BCG amplitude relative to left ventricle SV, during bike exercise with posture interventions.

We collected wrist accelerometry and radial artery arterial BP in healthy volunteers during a reclining bike exercise protocol. To address the low signal-to-noise (SNR) ratio of the wrist signal and the spectral instability of the systolic complex associated with AVO, we employed ensemble averaging and ensemble empirical mode decomposition (EEMD) to track the systolic complex of the BCG (Wu and Huang, 2011). We calculated BCG-PPG PTT and systolic BCG peak prominence across four discrete exercise levels in supine and semi-recumbent postures. Additionally, Doppler echocardiography of blood flow velocity in the left ventricle outflow tract (LVOT) confirmed the association of the wrist BCG signal with AVO and indicated a positive SV relationship with wrist BCG amplitude for a subset of participants with usable SV data. By examining wrist accelerometry and radial artery arterial BP during a reclining bike exercise protocol, we contribute to the understanding of wearable ballistocardiography and its potential applications in cardiovascular monitoring.

## 2 Materials and methods

### 2.1 Study protocol

#### 2.1.1 Participants

The study protocol was reviewed and preapproved by the Regional Committee for Medical Ethics Central Norway, application number 62226. The use of an experimental medical device was approved by the Norwegian Medicines Agency (reference 21/06743). The study was registered with ClinicalTrials.gov with identifier NCT05008133.

Twenty-five healthy volunteers aged between 21–43 were recruited via public advertisement in Trondheim, Norway. Exclusion criteria were reduced manual circulation, diabetes, known cardiovascular disease or increased thrombogenic risk. Screening was conducted by a trained anesthesiologist and all participants provided written informed consent. A summary of participant demographics is presented in Table 1.

**TABLE 1:** Participant demographics.

	All (*n* = 25)	Males (*n* = 12)	Females (*n* = 13)
Age, years	32 (21–43)	28 (21–39)	37 (26–43)
Height, cm	174 (154–193)	179 (173–193)	163 (154–177)
Weight, kg	70 (52–92)	78 (70–92)	62 (52–82)
BMI	23.5 (18–27)	24.0 (22–26)	22.9 (18–27)
Handedness			
Right	24 (96%)	13 (100%)	12 (92%)
Left	1 (4%)	—	1 (8%)
bSBP, mmHg	124 (103–147)	129 (112–147)	120 (103–137)
bDBP, mmHg	81 (55–106)	82 (70–106)	81 (55–95)

Values are median with absolute ranges in brackets. BMI: body mass index, bSBP: brachial systolic blood pressure, bDBP: brachial diastolic blood pressure.

#### 2.1.2 Device placement

**FIGURE 1:**
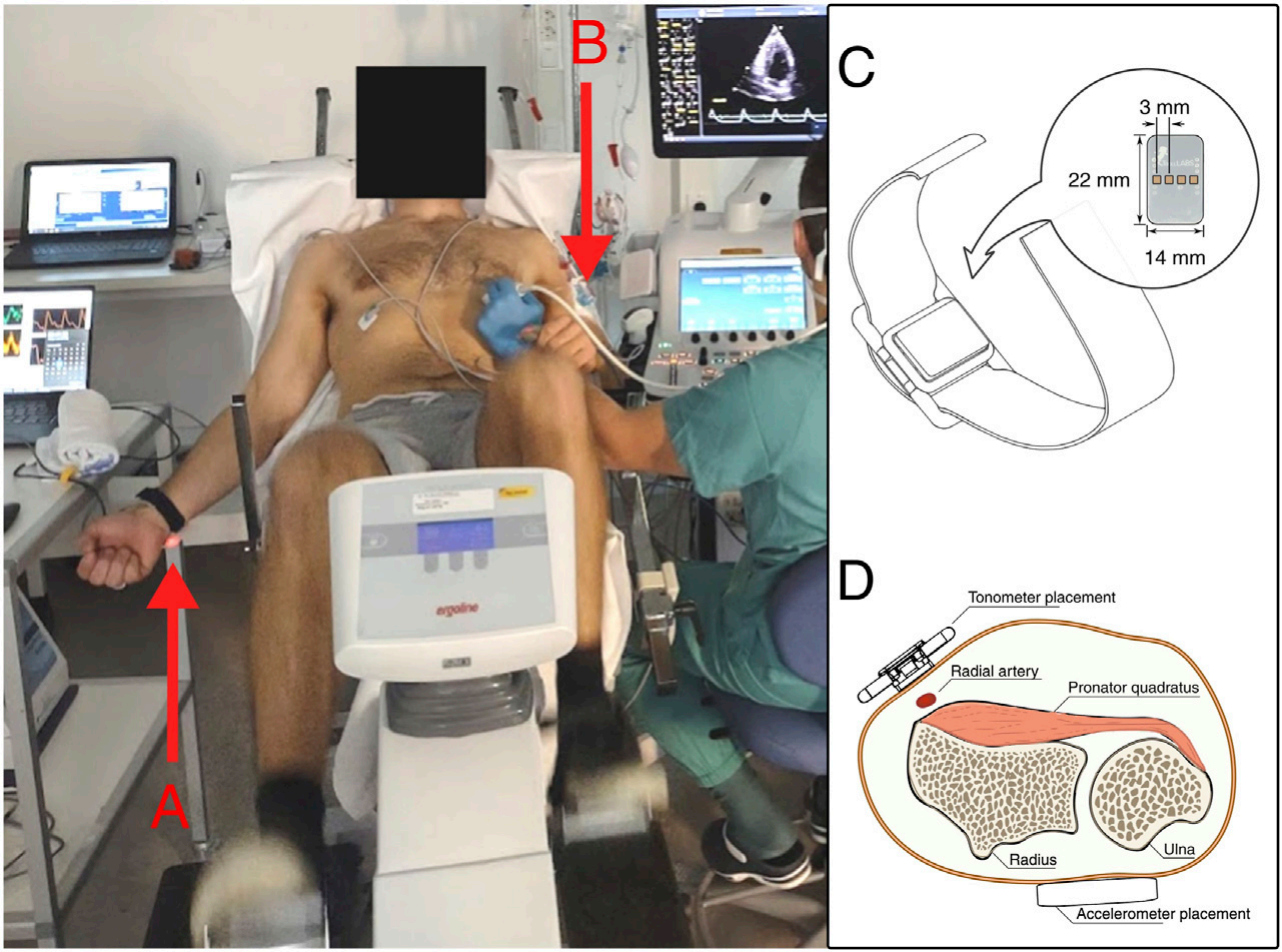
Left: Setup during semirecumbent sampling. **(A)** The investigational device is visible on the participant’s right wrist. **(B)** The pressure transducer is fixed to the upper non-dominant arm, which is rested on the handle of the recumbent bike. Photo by written consent. Right: the investigational device. **(C)** Illustration of the tonometer wristband. **(D)** Sensor placement on the dominant arm.

The investigational device consisted of a wristband-mounted sensor unit, composed of a microcontroller, accelerometer, galvanic skin response electrodes and an array of pressure sensors for radial tonometry. Data from the device was recorded to PC via wired serial communication. The investigational device was fitted to the participant’s wrist on the dominant arm (Figure 1). The arm hanged freely during exercise and relaxed on a bedside table during rest. Data from the sensors were recorded to a PC via wired serial communication.

#### 2.1.3 Acceleration ballistocardiography

A 3-axis MEMS accelerometer (LISD3H, STMicroelectronics, Switzerland) embedded in the investigational device was used to record acceleration in the dominant wrist. The accelerometer was located on the posterior of the wrist, like a large wristwatch (Figure 1). The investigational device’s output was recorded to PC via wired serial communication.

#### 2.1.4 Tonometry

A wearable tonometer integrated in the same wristband was placed above the radial artery immediately below the wrist of participants’ dominant hand. The device consisted of a linear array of silicone polymer-covered MEMS pressure sensors (BMP390, Bosch Sensortec Gmbh, Germany) to increase the viable placement area (Solberg et al., 2019).

#### 2.1.5 Photoplethysmography

The photoplethysmogram is obtained by measuring the intensity of specific wavelengths of reflected light in the vascular bed of well-perfused tissues. Transmission thumb PPG was recorded via the patient monitor and recorded to PC.

#### 2.1.6 Doppler echocardiography

Continuous wave Doppler imaging was used to record local flow velocity in the left ventricle outflow tract (LVOT) (Vivid e95, GE Vingmed, Norway). Together with flow velocity, LVOT diameter was recorded to allow estimation of left ventricle stroke volume via the velocity-time integral.

#### 2.1.7 Electrocardiography

3-lead ECG was recorded via the ultrasound machine.

#### 2.1.8 Arterial blood pressure

Arterial blood pressure was recorded in the radial artery via arterial line transducer (Meritrans DTXPlus, Merit Medical Systems, United States). The radial artery of the non-dominant arm was cannulated following standard clinical procedure under ultrasound guidance. The transducer was fixed on the upper arm at the midaxillary line and zeroed to atmospheric pressure. The transducer output was input to the patient monitor (Intellivue MX800, Philips, Netherlands) and recorded to PC from the monitor at a recorded output data rate of 125 Hz. The transducer was re-zeroed between postural changes.

### 2.2 Data collection and preprocessing

#### 2.2.1 Sampling protocol

**FIGURE 2:**
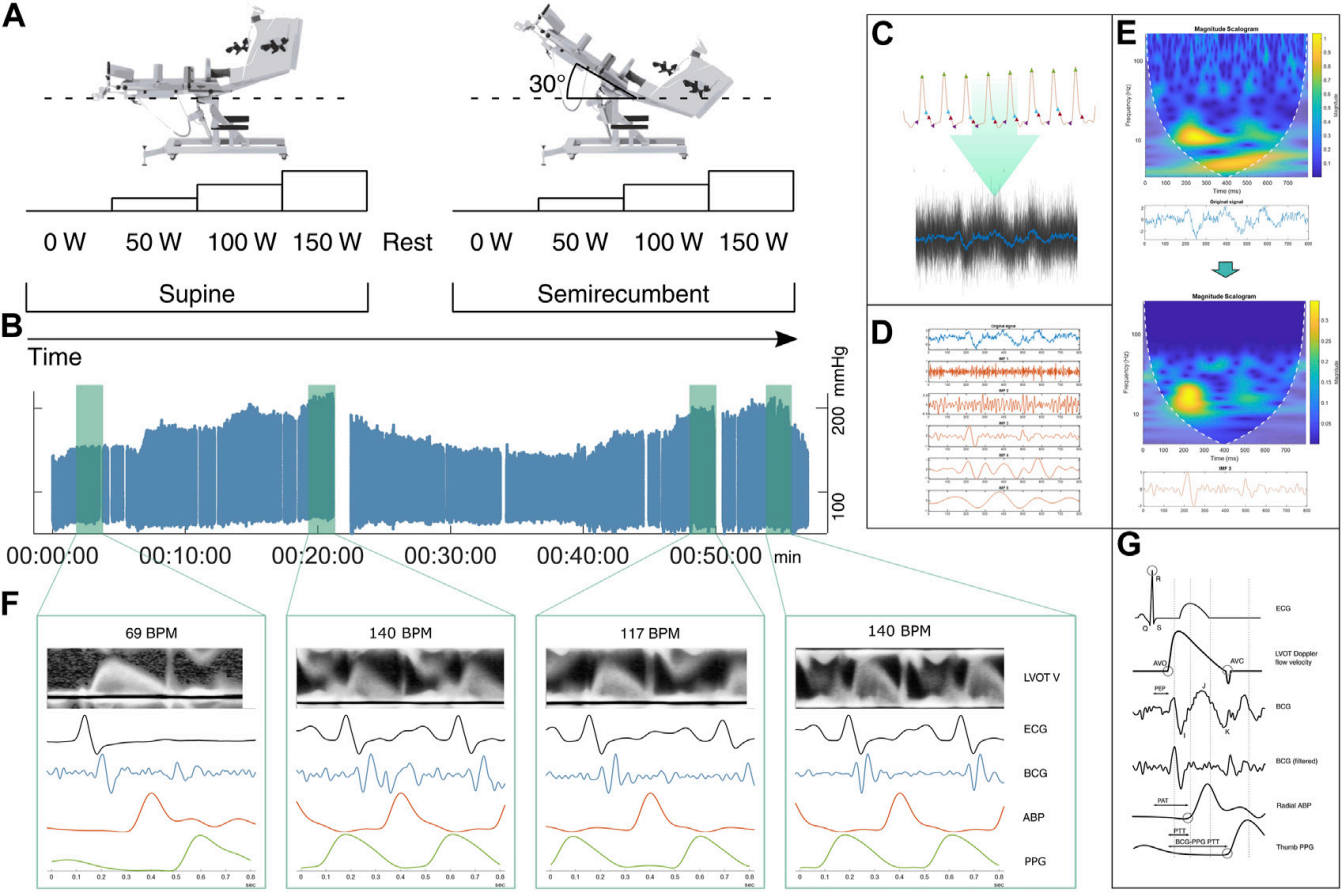
Graphical overview of data collection and data processing pipeline. **(A)** Schematic representation of exercise protocol. Each participant exercised freely at four different load levels from 0 to 150 Watts as defined by work delivered to the reclining ergometer bike, set via programmable dynamic resistance at 60 RPM. The ramping protocol was repeated for supine and semirecumbent posture with a rest and recovery period between. Rest took place in the semirecumbent posture. **(B)** Plot of ABP for one participant over the full session, roughly vertically aligned with the respective load states. **(C)** Raw accelerometer data is segmented into individual beats using systolic peaks as reference. Several segments form an ensemble average. **(D)** The average signal is decomposed via EEMD. **(E)** Time-frequency representation of the ensemble average and one of the IMFs. Top: The original signal. Bottom: Processed signal, emphasizing energy in the 10–30 Hz band. A temporally localized energetic feature appears to be isolated around the 200 ms mark. **(F)** Ensemble averaged signals between various exercise conditions, showing differences in wave morphology. Plotting the filtered BCG average against the ensemble averaged LVOT doppler flow images, ECG, and ABP, shows the correspondence of the identified high frequency BCG complex with the onset of rapid ejection. **(G)** Schematic diagram of the timing between signal features, illustrating the relationship between pre-ejection period (PEP), pulse arrival time (PAT), and pulse transit time (PTT). ABP: arterial blood pressure in radial artery, AVO: aortic valve opening, AOC: aortic valve closure, BCG: ballistocardiogram, ECG: electrocardiogram, EEMD: ensemble empirical mode decomposition, IMF: intrinsic mode function, LVOT-V: Left ventricle outflow tract velocity.

The sampling sequence consisted of two sets of ergometer exercise at four intensity levels. The first set was performed with the participant lying supine, followed by a resting period of about 15 min. The ergometer was then tilted back to a semi-recumbent position, with the participant reclining at a 30° angle. The arterial line was re-zeroed between postures. Exercise intensity was measured in Watts delivered to the ergometer and controlled via programmable dynamic resistance. The exercise levels were 0, 50, 100, and 150 W, tilted in four increments. The experimental protocol and the test environment are shown in Figure 2. Participants were instructed to maintain as close to 60 RPM as possible, which they could self-monitor via an integrated display. Participants exercised freely at each resistance level until HR and BP stabilized before measurements were taken. The pre-measurement period was at least 2 min followed by at least 2 min of measurements.

#### 2.2.2 Timing signal

To allow synchronization of disparate signals, an electronic timing signal was generated by a PC running a custom MATLAB script. This signal was recorded across the recording PC, the ultrasound machine, and the investigational device. All signals were resampled to a common frequency and synchronized using the timing signal using R (R Core Team, Austria). The BP and PPG signals were recorded at 125 Hz, the ECG was recorded at 500 Hz, the tonometer at 200 Hz, and the accelerometer was sampled at 900 Hz.

#### 2.2.3 Segmentation and ensemble averaging

BCG recorded from the wrist is noisier and more morphologically smooth than the full-body scale BCG, complicating identifying pertinent features in the signal. A common method to improve the signal to noise ratio is to take the ensemble average of multiple signal periods (Wiens et al., 2017). Figure 2 illustrates the processing pipeline from raw accelerometry data as described below.

Data from each sampling state was segmented using the systolic peak of the tonometry signal as a reference. To retain the systolic complex of the BCG and the foot of the following PPG signal, each segment was defined as 400 ms before and after the systolic peak of the accompanying radial pulse wave. Segmenting the signals to a known time range rather than from systole to systole simplified further analysis by having a common length of the signal vectors. To identify the BCG, the accelerometer axis corresponding to the head-foot axis was selected.

#### 2.2.4 Signal decomposition

For each sampling condition, the mean of the segmented beats was calculated. This ensemble average was then decomposed using EEMD. EEMD is a noise-assisted data-adaptive method which has proven to be effective at decomposing noisy biological signals with unstable spectral components, such as BCG signals (Enayati, 2019). It decomposes a signal into so-called intrinsic mode functions (IMF), forming a basis from which the original signal can be reconstructed. The EEMD prioritizes signal features that are AM-FM modulated, implying retention of physically significant modes.

**FIGURE 3:**
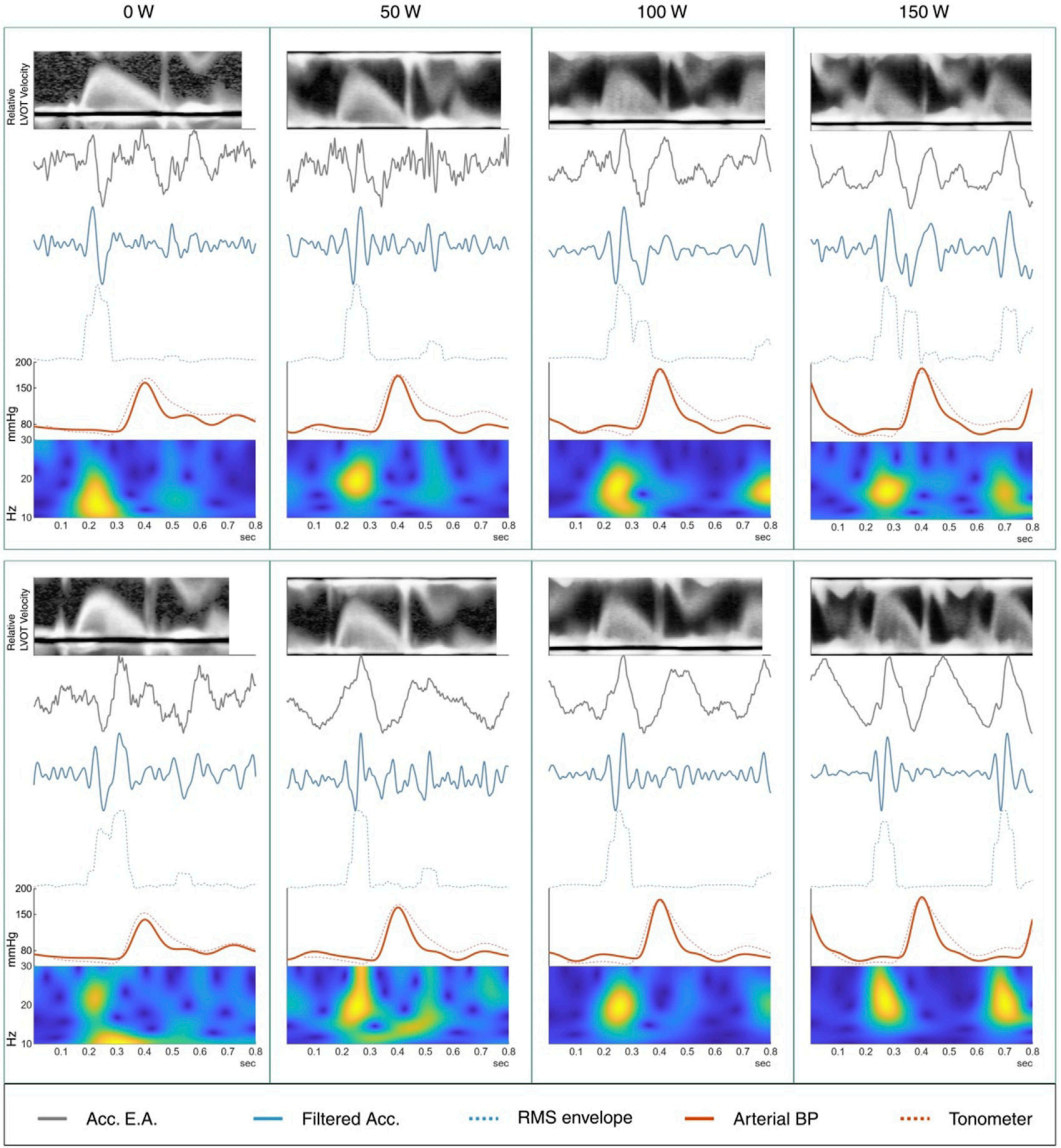
Ensemble averages from a male participant across all 8 exercise conditions. Top row is supine; bottom row is semirecumbent. At top, the averaged left ventricle outflow tract (LVOT) flow velocity shows onset of rapid ejection around 0.15–0.2 s followed by the “click” of the aortic valve closing at approximately 0.3–0.45 s. Below, the raw ensemble average of the accelerometer, followed by the filtered accelerometer signal with RMS envelope of the signal energy. Averaged arterial blood pressure (ABP) in the radial artery is shown together with the average tonometer signal, scaled to the magnitude of the ABP signal. Bottom: analytic morlet scalogram of the ensemble averaged accelerometer signals between 10 and 30 Hz. The sharp feature in the scalograms between 0.2 and 0.3 s seems to be clearly represented in the filtered BCG and is tentatively associated with rapid ejection from the ventricle.

To assess performance of the EEMD, we compute wavelet scalograms to visualize the localization of signal power across the frequency spectrum. The wavelet transform is especially suited to identifying local transients in the time domain, and is suited for BCG analysis (Malik and Boger, 2021). Figure 2E shows scalograms from the ensemble average of unfiltered accelerometer traces from one participant, as well as a selected IMF of the same signal. The original signal appears to contain two major components, one in the 10–30 Hz region tightly localized in time, and a larger scale, more smeared-out component in the 1–10 Hz band. The higher frequency components are isolated in IMF #3, consisting of a significant complex of deflections between 200–300 ms (100–200 ms prior to the radial systolic peak). Identifying this as the systolic complex, a later, smaller complex can be seen about 250 ms later, tentatively identified as the diastolic complex. We identify the higher-frequency component isolated in IMF #3 as the complex associated with AVO in the work by Wiens et al. (2017); Figure 3 further shows scalograms of acceleration ensemble averages across the exercise protocol, alongside the decomposed signal and the root-mean-square (RMS) envelope of the filtered signal’s power.

#### 2.2.5 Determining PTT and BCG amplitude

Pulse transit time was determined as the time between the onset of the systolic complex in the ballistocardiogram and the foot of the following PPG systole as determined using the intersecting tangents method. The reference point signifying the onset of the BCG systole was defined as the peak immediately preceding the first major negative deflection in the EEMD-filtered BCG. The amplitude was determined as the prominence of the largest peak within 120 ms of the onset of BCG systole.

Figure 3 shows the ensemble averages of BCG and simultaneously recorded ECG, radial artery BP, and LVOT velocity. Time-frequency scalograms show signal magnitude concentrations in the time window between the ECG R-peak and the foot of the pressure pulse wave in the radial artery. In comparison to the LVOT Doppler velocity curves, the onset of the signal complex occurs soon after the onset of rapid ejection.

Doppler flow velocity recordings were automatically traced using a custom MATLAB script. Tracings were manually inspected and corrected when necessary. By integrating the flow velocity from onset of ejection to valve closing, SV can be calculated by multiplying the resulting velocity time integral by the area of the LVOT. SV estimates were obtained by multiplying the velocity time integral by the LVOT diameter as measured in the ultrasound B-mode view.

#### 2.2.6 Hypothesis testing

Hypothesis testing was conducted using the two-sided Wilcoxon signed-rank test for paired data. The test calculates a test statistic describing the likelihood of the null hypothesis that the difference between two paired samples is from a distribution whose mean is zero. In each test case, observations from each participant in one condition were paired with the same participants’ measurements in the comparison condition. In case of missing observations, the participant was excluded. The significance criteria for rejecting the null hypothesis of no difference between sample populations was set to *a* = 0.05. Correlation strengths between variables was compared using the cocor R package with dependent groups at *a* = 0.05 (Diedenhofen and Musch, 2015). Post-hoc comparison of group effects but not interaction effects was supported by a non-parametric factorial analysis using the Aligned Rank Transform (Supplementary Table S1; Wobbrock et al., 2011).

## 3 Results

**FIGURE 4:**
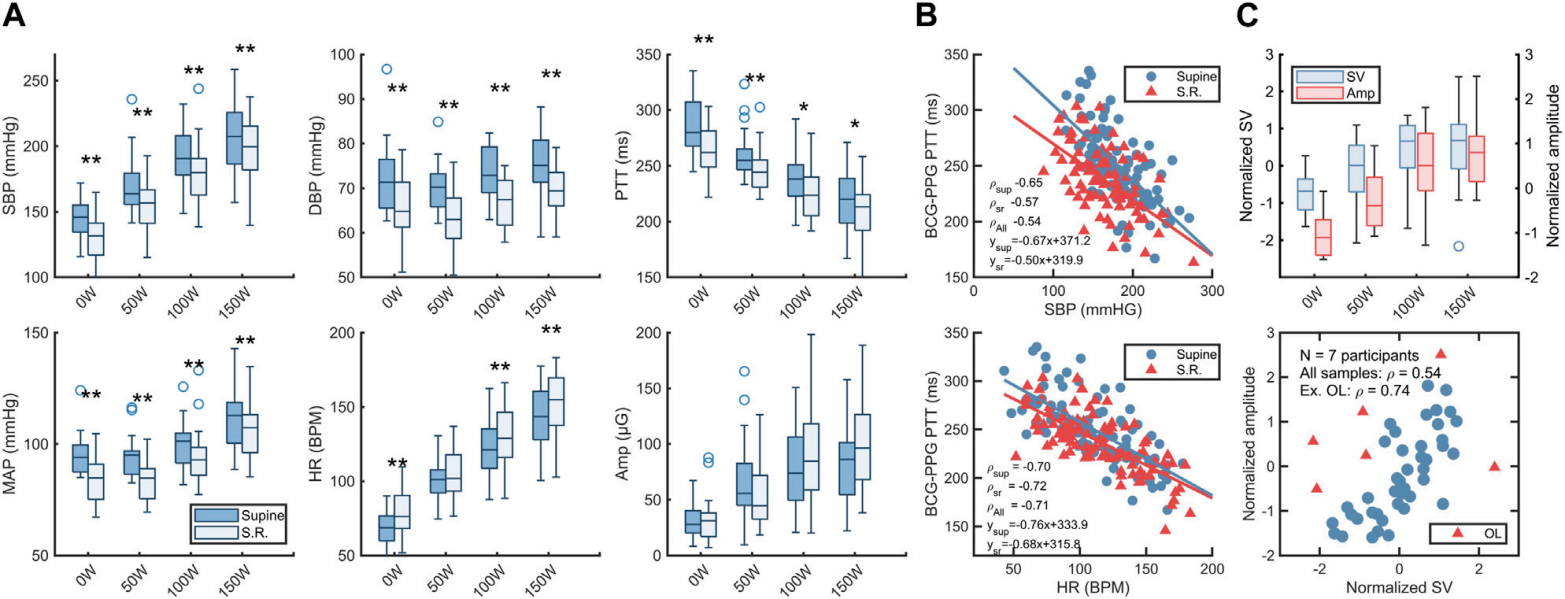
Hemodynamics between exercise states. **(A)** box charts showing distribution of systolic blood pressure (SBP), diastolic blood pressure (DBP), mean arterial pressure (MAP), heart rate (HR), BCG-PPG pulse transit time (PTT) and systolic BCG amplitude. Circles are outliers. Stars indicate significance of difference between postures via two-tailed Wilcoxon signed rank test at two different significance levels: **: *a* < 0.01, *: *a* < 0.05. **(B)** Top: scatterplot of SBP vs. BCG-PPG PTT with least-square regression lines. Bottom: scatterplot of BCG-PPG PTT vs HR. ρ is Pearson’s correlation coefficient. **(C)** Top: bar chart of normalized left ventricle stroke volumes *versus* BCG amplitude across exercise conditions. Bottom: scatterplot of systolic BCG amplitude *versus* left ventricle stroke volume (SV). SV data was only available from a subset of participants. OL indicates outliers determined via the robust Mahalanobis distance at a chi-square significance level of 0.05. SV and amplitude data were normalized by z-scoring across the entire sample.

Aggregate results are shown in Table 2 and Figure 4. Values are reported as mean with range except where otherwise noted.

**TABLE 2:** Summary of results.

Intensity	0 W		50 W		100 W		150 W	
Posture	Supine	S.R.	Supine	S.R.	Supine	S.R.	Supine	S.R.
HR, bpm	69.0 ± 12.0	79.1 ± 15.4	101.2 ± 13.9	104.0 ± 16.4	123.0 ± 18.0	130.1 ± 20.8	144.0 ± 19.3	151.8 ± 20.6
SBP, mmHg	145.3 ± 15.8	129.6 ±19.1	170.5 ± 22.0	154.3 ± 20.6	194.1 ± 26.3	179.8 ± 23.4	207.7 ± 27.0	200.9 ± 29.5
DBP, mmHg	71.8 ± 7.4	65.2 ± 7.4	70.3 ± 5.7	63.4 ± 6.1	73.2 ± 6.0	66.8 ± 5.7	75.0 ± 6.9	69.2 ± 5.6
MAP, mmHg	95.2 ± 8.9	83.4 ± 9.6	93.7 ± 8.9	83.5 ± 8.7	101.7 ± 14.2	93.4 ± 12.7	111.3 ± 13.9	107.7 ± 16.5
PTT, ms	286.2 ± 25.2	263.1 ±23.6	260.0 ± 21.5	246.0 ± 30.3	237.8 ± 21.1	227.3 ± 23.2	219.6 ± 26.1	209.1 ± 27.8
Amp, µG	29.5 ± 15.2	31.7 ± 20.4	65.4 ± 37.8	52.3 ± 27.2	78.8 ± 35.8	88.6 ± 39.5	82.2 ± 38.0	98.2 ± 37.8

Data are mean ± one standard deviation. Amp, systolic amplitude of filtered BCG, signal; DBP, diastolic blood pressure in radial artery; HR, heart rate; MAP, mean arterial pressure in radial artery; PTT, BCG-PPG, pulse transit time; SBP, systolic blood pressure in radial artery; S.R., Semirecumbent. blood pressure values are in the radial artery via arterial line.

### 3.1 Blood pressure during exercise

Systolic blood pressure increased with exercise load in both postures. Excluding two participants who interrupted the supine 150 W condition, the median increase from rest to 150 W was 62 (31.3–116.1) mmHg in the supine condition *versus* 60.7 (37.5–112.8) mmHg in the semirecumbent condition. Diastolic pressures varied less, with a median increase of 1.2 (−18.7–16.3) mmHg in supine *versus* 6.3 (−9.3–22.2) mmHg semirecumbent (Table 2). Systolic, diastolic, and mean arterial blood pressure was consistently lower in the semirecumbent posture than in the supine posture (Figure 4).

### 3.2 Heart rate

Heart rate increased linearly with exercise load, with similar increases between supine and semirecumbent conditions. Heart rate was slightly higher in the semirecumbent posture, but the difference was not consistently significant (Figure 4).

### 3.3 BCG-PPG PTT

PTT decreased with exercise load in both supine and semirecumbent states (Table 2; Figure 4). Semirecumbent PTT was significantly lower than supine throughout, although the difference became less clear with increasing workload (*p* < 0.001 for 0 and 50 W, p_100W_ = 0.0135, p_150W_ = 0.031).

PTT exhibited a moderate negative correlation with radial SBP (Figure 4). The correlation was strength was slightly elevated in the supine condition compared to the semirecumbent, as well as the total (ρ_Sup_ = −0.65 vs. ρ_SR_ = −0.57, ρ_All_ = −0.54), but this difference was not statistically significant. PTT had a clear negative correlation with increasing HR (ρ_All_ = −0.71), but with little distinguishable difference between postures (Figure 4).

### 3.4 BCG amplitude

Systolic amplitude in the BCG signal increased significantly from rest at 0 W (32.4 ± 18.5) to 50 W (65.4 ± 37.8) (*p* = 0.002) and from 50 to 100 W (86.8 ± 38.0) (*p* < 0.001) (Table 2), but the difference between 100 and 150 W was not significant (*p* = 0.24). There was wide variation between participants, but the mean amplitude increased threefold between rest and 150 W in both postures, reaching close to maximum value at 100 W. There was not a significant difference in amplitude between supine and semirecumbent exercise across all conditions or between individual conditions.

### 3.5 Stroke volumes

The LVOT Doppler velocity recordings during exercise were characterized by large amounts of noise, complicating VTI calculation. A subset of 7 participants with relatively clean recording series were selected and stroke volumes calculated via LVOT VTI. Normalized SV data for both postures is plotted against BCG amplitude in Figure 4, section C In this small subsample, BCG amplitude increases with SV (ρ = 0.54). Excluding outliers according to robust Mahalanobis distance estimates (Leys et al., 2018), the correlation across all sample conditions increased to ρ = 0.74 (Figure 4). Excluding outliers could be motivated by presupposing low data quality.

## 4 Discussion

### 4.1 AVO detection

Comparing the averaged and filtered BCG signals with the Doppler velocity in the LVOT shows a high frequency BCG complex occurring shortly after the onset of rapid ejection, identified by the sudden rapid increase in flow velocity in the LVOT (Figure 3). This temporal relationship continues with increasing heart rate. The initial deflection of the higher-frequency bands of the wrist BCG, around 10–40 Hz, seems to reliably mark the beginning of ventricular ejection to within a few milliseconds. It is notable that the increase in BCG amplitude with increasing exercise load may make the features of interest easier to detect than during rest or low activity, when the small signal amplitude can get lost in noise and even small motion artifacts.

Using BCG as a proximal marker of AVO and onset of ejection has been demonstrated using full-body force plate BCG, for example, by Inan et al. alongside LVOT echocardiography (Inan et al., 2009a). Wiens et al. further examined the use of wrist BCG to mark AVO (Wiens et al., 2017).

### 4.2 Amplitude

The amplitude of the systolic complex increased rapidly from rest to 50 and again from 50 to 100 W but leveled off between 100 and 150 W. While increases from 0 to 50 and 50–100 W were each significant (*p* < 0.01), there was no statistically significant difference between 100 and 150 W in either exercise condition. This is consistent with the hypothesis that the energy content (e.g., amplitude and peak width) of the BCG is closely related to SV, which is expected to increase linearly with HR until plateauing as ventricular filling capacity is reached. Further increase in CO then becomes primarily driven by an increase in HR. Indeed, the same leveling off at 100–150 W appears in the limited SV data available (Figure 4). Systolic amplitude corresponding to SV makes intuitive sense if interpreted as a marker of the mechanical energy delivered into the cardiovascular system per heartbeat (Inan et al., 2015). The relationship between mechanical energy in BCG and similar systems and SV, together with systolic timing intervals, has been exploited for assessment of markers of cardiovascular function such as cardiac output and oxygen uptake (Inan et al., 2009a; Shandhi et al., 2021).

Amplitude did not differ significantly between supine and semirecumbent posture. The large variance in the collected data may hide any significant differences, although it is acknowledged that BCG morphology varies substantially between individuals, reflecting differences in vascular anatomy and body composition (Starr, 1955). This variation complicates detailed morphological analysis.

Difference in coupling between the mass of the body and the supporting surface has been proposed as a contributor to posture variability in the wrist ballistocardiogram morphology (Shin et al., 2022). In this study, participants were lying on the same recumbent bicycle for the duration of the test protocol. As such the difference in mechanical coupling should be lessened between postures, although the action of the center of mass changed.

### 4.3 Postural effects

#### 4.3.1 PTT

We observed significantly lower PTT in the semirecumbent state. Our findings are consistent with Shin et al. who noted significant differences in BCG-PPG PTT recorded at the wrist between upright and supine postures at rest (Shin et al., 2022). In their study, supine PTT was approximately ∼20 ms higher than standing and sitting, which were not significantly different. This is in line with our observations for PTT at rest and during mild exercise (Figure 4). At higher intensities of exercise (100 and 150 W) the difference in PTT was less pronounced.

Both changes in the distending pressure in the arteries and sympathetic nerve action cause changes in arterial elasticity during dynamic exercise (Agnoletti, 2018). We observed a slight, but inconsistent increase in HR between supine and recumbent postures, while MAP remained lower in the semirecumbent position. Lefferts et al. also noted increases in arterial stiffness following acute exercise, which may be a separate confounder in our data as the rest period between each postural condition was not very long (Lefferts et al., 2018).

The difference in systolic BP and MAP between supine and semirecumbent situations may go some way to explain the disparity in PTT at rest and at light exercise. However, arterial stiffness measured via pulse wave velocity has been reported to increase between supine and semi-upright upper-body position independent of significant changes in height-corrected BP (Schroeder et al., 2017). The exact cause of this disparity in PTT between postures seems unclear. In the case of wrist based BCG measurement, Shin et al. propose hydrostatic effects between measurement sites in the upper and lower arm (Shin et al., 2022), but as we recorded BP in the lower arm we would expect to see a consistently higher BP in the semirecumbent state if the decrease in PTT were to be driven by increased BP in the peripheral limb, which is the opposite of what we observed.

#### 4.3.2 Blood pressure

While SBP, DBP and MAP all increased with exercise intensity, BP was consistently and significantly higher in the supine posture. Part of the observed effect could be influenced by exercise-induced hypotension in the semirecumbent condition, given the short rest period between postures (Carpio-Rivera et al., 2016), but increased upper limb BP in the supine condition as compared to sitting or semi-upright postures has been previously demonstrated (Netea et al., 2003; Watanabe et al., 2007). In the context of ergometer exercise, decreased gross efficiency in the supine condition compared to recumbent exercise state at 70 W has been shown, hypothesized to be primarily due to height differences between the heart and the working muscles in turn affecting cardiovascular performance during exercise (Wehrle et al., 2021).

### 4.4 Study limitations

The low signal-to-noise ratio of the acceleration measurements makes continuous beat-to-beat recording of the systolic complex difficult. An inexpensive accelerometer with a noise floor of 220 µg/ $\sqrt{Hz}$ was used. A low accelerometer noise floor has been reported to improve signal quality in wearable applications (Shandhi et al., 2021). The use of ensemble averages is predicated on the assumption that the signals are locally stationary, which limits the applicability of this approach as HR varies dynamically with exercise. Accelerometers with lower noise floors may allow smaller ensembles to be averaged to produce a usable signal in more dynamic exercise use cases.

A weakness of the present study lies in the use of steady state exercise. Steady exercise conditions allow multiple heartbeats to be averaged, mitigating signal quality issues to a degree. This approach however removes some of the variance in the averaged data (breathing cycles, for instance) which may be of interest. The steady states examined here may furthermore not be directly comparable to many uncontrolled exercise conditions where exertion levels might vary more dynamically. Application in more realistic exercise situations requires further work in instrumentation and signal processing to handle motion artifacts and enable the use of shorter ensemble windows. The sample size of the current study is in any case small, and extrapolation to larger populations may also be complicated by the homogenous makeup of the participant group.

The four exercise load levels–set to absolute levels of delivered work–allow for intra-individual comparison across a linearly increasing load, but individual response at each level will vary significantly. In the absence of quantitative measures of perceived exertion, i.e., Borg scale ratings, inter-individual comparisons must be made with caution.

Segmentation of the signal using BP waveforms offers interesting opportunities in combination with wearable tonometry. ECG, often used for waveform segmentation, is difficult to acquire at the wrist in a passive scenario, requiring extra leads or intentional contact to another body part. Similarly, the PPG waveform is often morphologically smooth, making consistent determination of reference points challenging. With adequate contact force, radial tonometry could provide a reliable segmentation reference for wearable applications.

## 5 Conclusion

Acceleration ballistocardiograms may be recorded at the wrist even during relatively high exercise loads. A secondary sensor mode acting as a reference allows segmentation of the signal and the creation of ensemble averages, from which timing references closely following AVO can be obtained at significantly elevated HR. By combining the BCG-derived proximal cardiac reference with a reference of the arrival of a peripheral pulse wave, estimates of PTT may be calculated in this way.

PTT decreased with increasing exercise load, closely following HR trends. Like both systolic, mean, and diastolic arterial blood pressure, PTT was lower in the semirecumbent posture, while HR was higher in this state. Consequently, while PTT showed clear correlation with both systolic BP and HR, not all the variance in PTT may be explained by increase in HR alone.

Convenient PTT measurement during exercise may enable broader studies of cardiovascular response to acute exercise outside the lab setting, which in turn may facilitate more naturalistic monitoring of cardiovascular performance in persons with CVD risk.

## Data Availability

The raw data supporting the conclusion of this article will be made available by the authors, without undue reservation.
