# Supplementary material for: Wrist ballistocardiography and invasively recorded blood pressure in healthy volunteers during reclining bike exercise
Source: Front Physiol. 2023 May 12;14:1189732. doi: 10.3389/fphys.2023.1189732 (PMC10213206; doi:10.3389/fphys.2023.1189732)
Supplement: Supplementary file 1 [file Table1.DOCX]

Supplementary Material

Wrist ballistocardiography and invasively recorded blood pressure in healthy volunteers during reclining bike exercise

**Torjus L. Steffensen, Filip E. Schjerven, Hans M. Flade, Idar Kirkeby-Garstad, Emma Ingeström, Fredrik S. Solberg, and Martin Steinert**

*** Correspondence:** Torjus Steffensen: torjus.l.steffensen@ntnu.no

# ANOVA

Due to concerns of the data not fulfilling the necessary assumptions of calculating a standard repeated-measures ANOVA analysis, we opted to use the Aligned Rank Transform for doing a nonparametric factorial analysis (Wobbrock et al., 2011; Kay et al., 2021). ART allows for the testing of group and interaction effects, even in the case of repeated measurements, without assuming normality of residuals and with favorable properties in the case of slightly heterogenous variance within groups.

We used a mixed-effects model, with exercise intensity (0 – 50 – 100 – 150 Watts), posture (recumbent vs. supine), and their interaction as fixed effects, and a random effect accounting for repeated measurements performed on the same individuals. Individual entries where values were missing were excluded from the analysis, however, this was only the case of 1-3 individual datapoints per analysis.

The ANOVA analysis using ART was applied with systolic blood pressure (SBP), diastolic blood pressure (DBP), heart rate (HR), BCG amplitude, Pulse Transit Time (PTT), and mean arterial pressure (MAP) as dependent variables, separately, totaling six analyses. Significant group effects were detected for exercise intensity on all dependent variables (p < 0.0001). Significant group effects were detected for posture on all dependent variables (p < 0.0001) except the BCG amplitude (p > 0.05). No interaction effects were found significant for any dependent variable (Table 1).

| **Supplementary Table 1:  Group effects found significant in repeated measures ANOVA using ART** | | | |
| --- | --- | --- | --- |
| Dependent variable | Exercise intensity  (0 – 50 – 100 – 150 Watts) | Posture  (supine – semirecumbent) | Exercise intensity x position, interaction |
| SBP | * | * |  |
| DBP | * | * |  |
| HR | * | * |  |
| BCG Amp | * |  |  |
| PTT | * | * |  |
| MAP | * | * |  |

* signifies p < 0.05. All effects found to be significant were significant at p < 0.0001.

# References

Kay, M., Elkin, L. A., Higgins, J. J., and Wobbrock, J. O. (2021). mjskay/ARTool: ARTool 0.11.0. doi: 10.5281/zenodo.4721941.

Wobbrock, J. O., Findlater, L., Gergle, D., and Higgins, J. J. (2011). The aligned rank transform for nonparametric factorial analyses using only anova procedures. in *Proceedings of the SIGCHI Conference on Human Factors in Computing Systems* CHI ’11. (New York, NY, USA: Association for Computing Machinery), 143–146. doi: 10.1145/1978942.1978963.
